# Paramagnetism of cobalt-doped ZnO nanoparticles obtained by microwave solvothermal synthesis

**DOI:** 10.3762/bjnano.6.200

**Published:** 2015-09-30

**Authors:** Jacek Wojnarowicz, Sylwia Kusnieruk, Tadeusz Chudoba, Stanislaw Gierlotka, Witold Lojkowski, Wojciech Knoff, Malgorzata I Lukasiewicz, Bartlomiej S Witkowski, Anna Wolska, Marcin T Klepka, Tomasz Story, Marek Godlewski

**Affiliations:** 1 Institute of High Pressure Physics, Polish Academy of Sciences, Sokolowska 29/37, 01-142 Warsaw, Poland; 2 Institute of Physics, Polish Academy of Sciences, Al. Lotników 32/46, 02-668 Warsaw, Poland

**Keywords:** cobalt-doped zinc oxide, ferromagnetism, magnetic properties, microwave solvothermal synthesis (MSS), paramagnetism

## Abstract

Zinc oxide nanopowders doped with 1–15 mol % cobalt were produced by the microwave solvothermal synthesis (MSS) technique. The obtained nanoparticles were annealed at 800 °C in nitrogen (99.999%) and in synthetic air. The material nanostructure was investigated by means of the following techniques: X-ray diffraction (XRD), helium pycnometry density, specific surface area (SSA), inductively coupled plasma optical emission spectrometry (ICP-OES), extended X-ray absorption fine structure (EXAFS) spectroscopy, scanning electron microscopy (SEM), energy dispersive X-ray spectroscopy (EDS) and with magnetometry using superconducting quantum interference device (SQUID). Irrespective of the Co content, nanoparticles in their initial state present a similar morphology. They are composed of loosely agglomerated spherical particles with wurtzite-type crystal structure with crystallites of a mean size of 30 nm. Annealing to temperatures of up to 800 °C induced the growth of crystallites up to a maximum of 2 μm in diameter. For samples annealed in high purity nitrogen, the precipitation of metallic α-Co was detected for a Co content of 5 mol % or more. For samples annealed in synthetic air, no change of phase structure was detected, except for precipitation of Co_3_O_4_ for a Co content of 15 mol %. The results of the magentometry investigation indicated that all as-synthesized samples displayed paramagnetic properties with a contribution of anti-ferromagnetic coupling of Co–Co pairs. After annealing in synthetic air, the samples remained paramagnetic and samples annealed under nitrogen flow showed a magnetic response under the influences of a magnetic field, likely related to the precipitation of metallic Co in nanoparticles.

## Introduction

Nanomaterials have drawn the attention of researchers from all over the world due to their new, interesting perspectives in many application areas [1–3]. The most challenging issue nowadays is how to produce such advanced nanocrystals with well-defined and reproducible electronic, optoelectronic and magnetic properties at low cost.

In the present work we investigate zinc oxide (ZnO), which is an attractive material with a wide range of applications such as: transparent transistors based on semiconducting transparent oxides [4], ultraviolet (UV) light blockers [5], photocatalysts [6] or antibacterial uses [7]. The energy band gap of ZnO is ≈3.3 eV at room temperature, corresponding to a wavelength of about 375 nm [8]. Thus, ZnO has been reckoned as an excellent UV shielding material, characterized by photo-fastness and absorptivity over a broad UV range, in contrast to other organic and inorganic UV shielding materials [9]. Moreover, ZnO is applied to cosmetics [10], optoelectronic devices [11–14], solar cells [10], catalysts [15], energy storage (battery) materials [16], fast data storage [17], light-emitting diodes (LEDs) [18], gas sensors [10], thermoelectric devices [19], varistors [20–21], window materials for displays [21], laser technology [10], surface acoustic wave devices [22] and drug delivery [23].

Recently, zinc oxide doped with Mn, Co and Ni has been under investigation for potential application as a spintronic material [24–31]. Many experimental and theoretical studies of ZnO doped with transition metal (TM) elements suggest good prospects for TM:ZnO for use in diluted magnetic semiconductor (DMS) applications. A recent discussion regarding magnetic performance of these materials is focused on the homogeneity of the TM distribution in the host material and on the phase purity [32].

There are several ways to synthesize TM:ZnO DMS materials, including physical and chemical approaches. However, the physical methods suffer from difficulties in controlling thermal stability and the doping homogeneity in the final products [32–33]. The chemical approach has also been widely employed to synthesize ZnO-based DMS materials [32]. The applied techniques include: solvothermal [34–35], hydrothermal [36–37], sol–gel [38–39] among others. These techniques enabled the control of the size and morphology of the particles by setting appropriate conditions, for instance, synthesis temperature, time, and the concentration of precursors.

For wide application of Zn_1−_*_x_*Co*_x_*O in spintronics, a ferromagnetic response (FM) at room temperature (RT) is required. This was theoretically predicted [34–35] and there are claims of experimental confirmation of these predictions [40–42]. However, there were also a number of observations contradicting this prediction [34,36,38,43] and repeatability of results is a continuing problem [36]. Many papers report a paramagnetic response [36,42–45]. According to the literature, it can be conferred that the use of solvothermal synthesis allowed Co-doped ZnO to be obtained with both ferromagnetic and paramagnetic properties [46–47]. Using hydrothermal [32,48] and sol–gel [49–50] synthesis methods, Co-doped ZnO with both ferromagnetic and paramagnetic properties was obtained [39,41–42]. These observations questioned the possibility of producing powders with controllable properties suitable for practical application as a spintronic material.

Microwave activation of hydrothermal synthesis used to obtain nano-sized powders has been reported in a number of papers [51–57]. Microwave ovens were already vastly adopted in the 1980s for the synthesis of organic compounds [58–66]. The advantages of the method are, in addition to high purity synthesis conditions, a high heating range, precise time control, and the possibility to obtain fully reacted nanoparticles (which means no unreacted precursors or hydroxides remain on the nanoparticle surface) with a narrow grain size distribution. It has been proved that many reactions based on hydrothermal synthesis can be carried out with high efficiency and low energy consumption [67]. Doped nanoparticles can be produced using these methods as well [67]. Thus, we expected that fully reacted, Co:ZnO nanoparticles could be produced using microwave solvothermal synthesis (MSS) [68–71].

Magnetic investigations were performed to verify several points. First of all, we tested if uniform samples grown by a low temperature MSS process show paramagnetic response, as observed in ZnMnO systems [34]. Next, we investigated effects related to an increased Co concentration in the powders. Finally, we performed magnetic investigations for samples annealed under different gas environments, while keeping the same annealing temperature and process time. Another question was also the limit of Co content for paramagnetic behavior, and also, whether annealing of the synthesized powders will lead to Co clustering and the appearance of a FM response.

## Results and Discussion

### Nanopowder characterization

**Figure 1:**
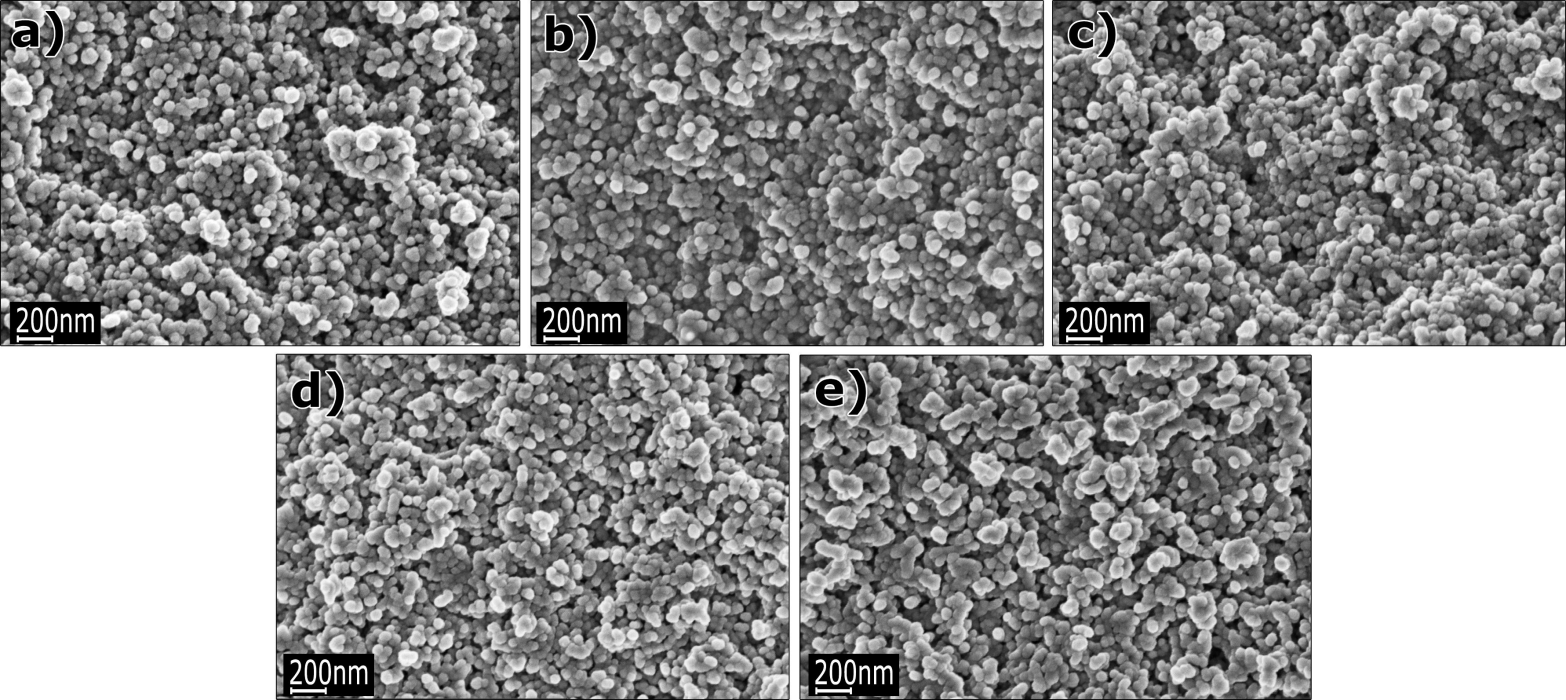
SEM images of ZnO nanopowders in their as-produced (before annealing) state: (a) undoped, (b) doped with 1 mol % of Co^2+^, (c) 5 mol % of Co^2+^, (d) 10 mol % of Co^2+^, and (e) 15 mol % of Co^2+^ ions.

**Figure 2:**
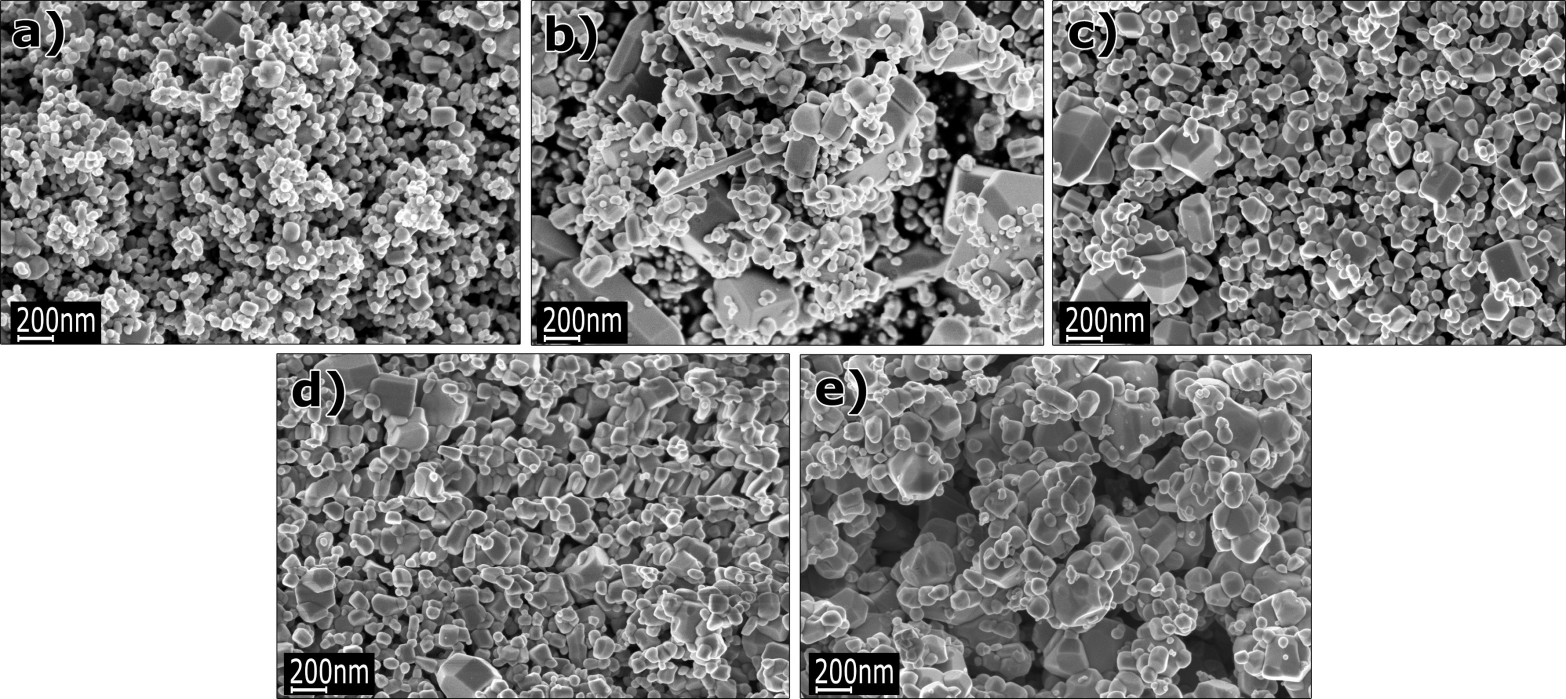
SEM images of annealed ZnO nanopowders after annealing at 800 °C in nitrogen: (a) undoped, (b) doped with 1 mol % of Co^2+^, (c) 5 mol % of Co^2+^, (d) 10 mol % of Co^2+^, and (e) 15 mol % of Co^2+^ ions.

**Figure 3:**
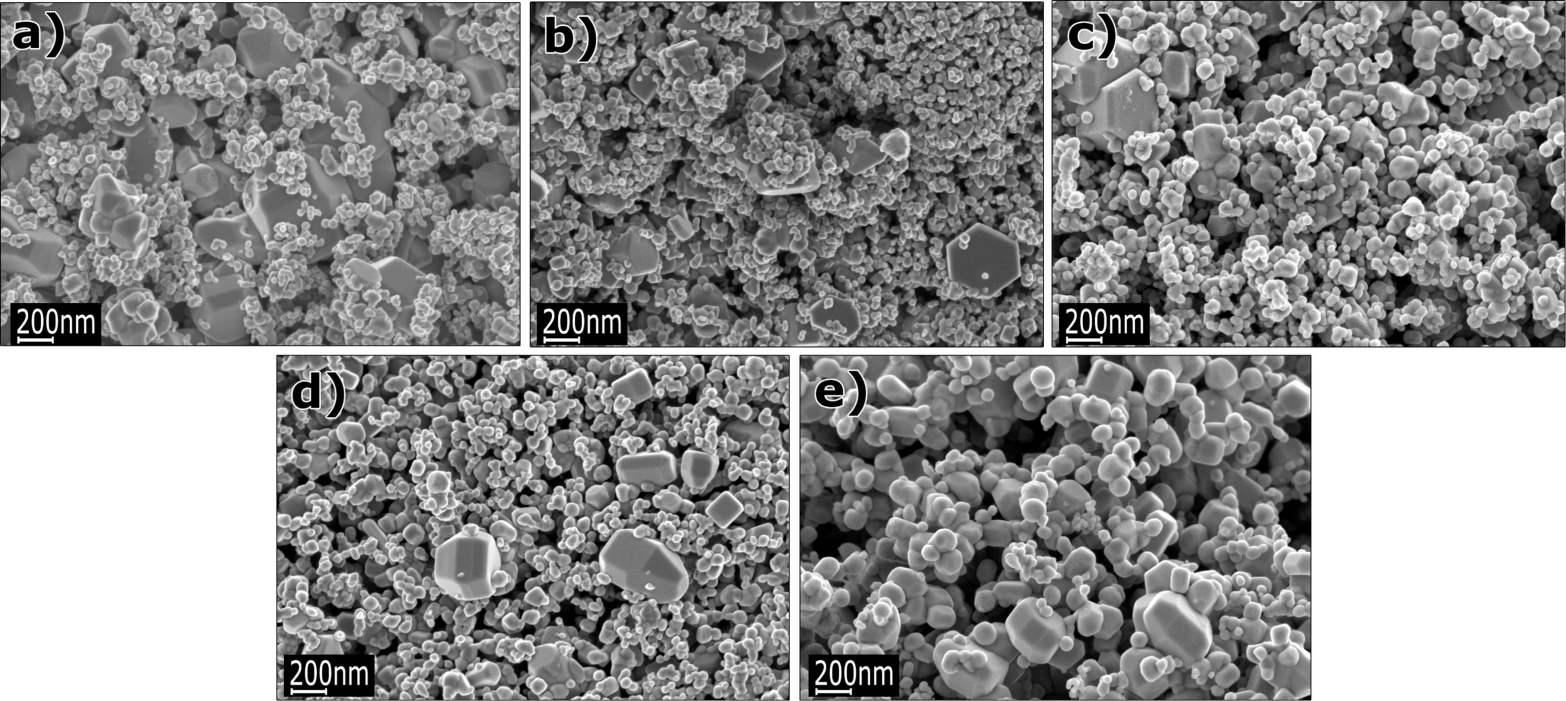
SEM images of annealed ZnO nanopowders after annealing at 800 °C in synthetic air: (a) undoped, (b) doped with 1 mol % of Co^2+^, (c) 5 mol % of Co^2+^, (d) 10 mol % of Co^2+^, and (e) 15 mol % of Co^2+^ ions.

SEM images of undoped and 1%, 5%, 10%, and 15% Co-doped ZnO nanopowders in their as-produced state (before annealing) and after heat annealing in nitrogen and synthetic air are presented in Figure 1, Figure 2 and Figure 3, respectively. According to the SEM investigations, the average particle diameter ranges from 30 to 70 nm (Figure 1). Figure 2 and Figure 3 show particles annealed at 800 °C under a nitrogen and synthetic air environment, respectively. The particle size after annealing ranges from 30 nm to 2 µm and depending on the amount of Co, the particle shape has a tendency to change from spherical to hexagonal or cubic.

**Figure 4:**
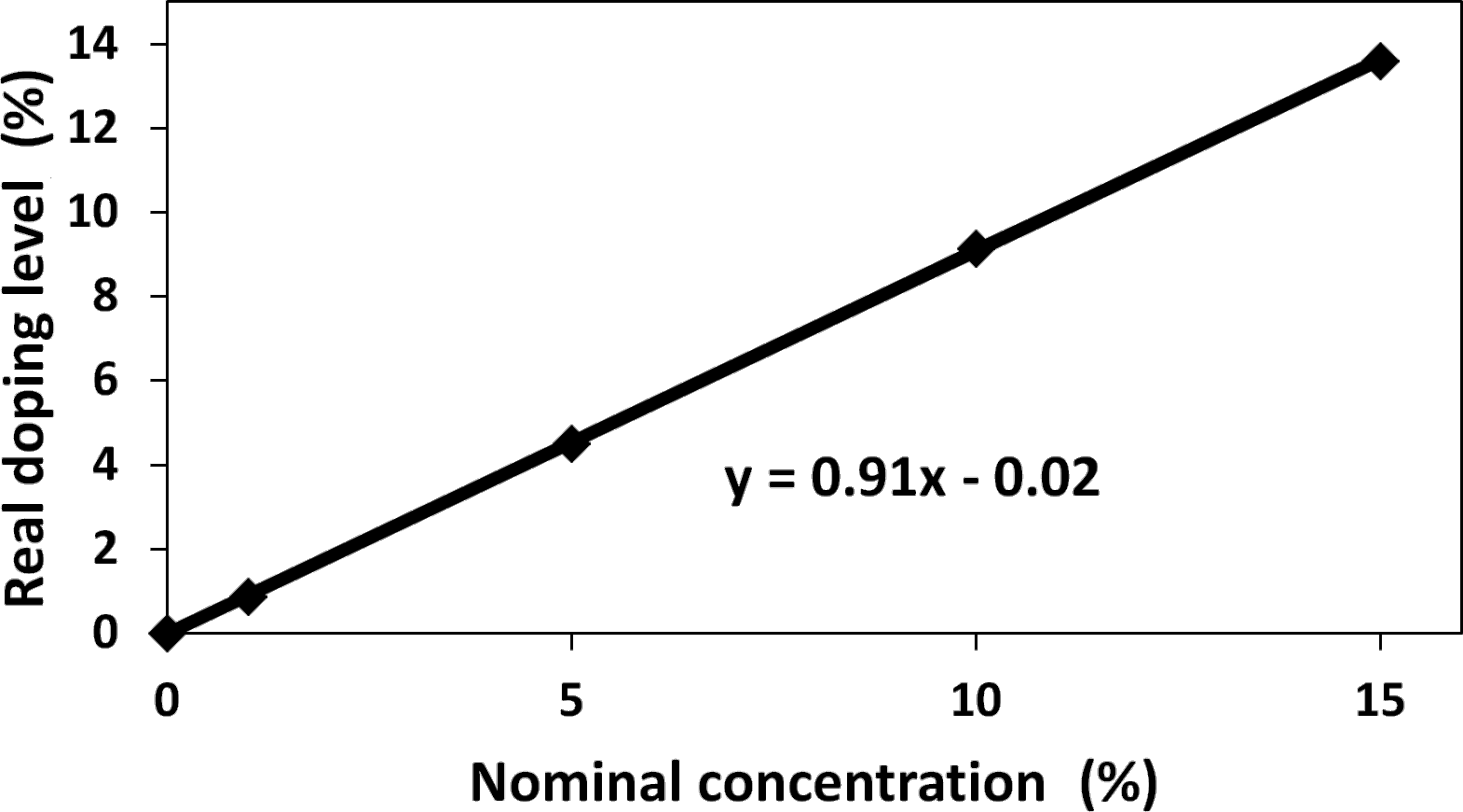
Dependence between the nominal and experimental Co doping concentration for the samples before annealing.

**Figure 5:**
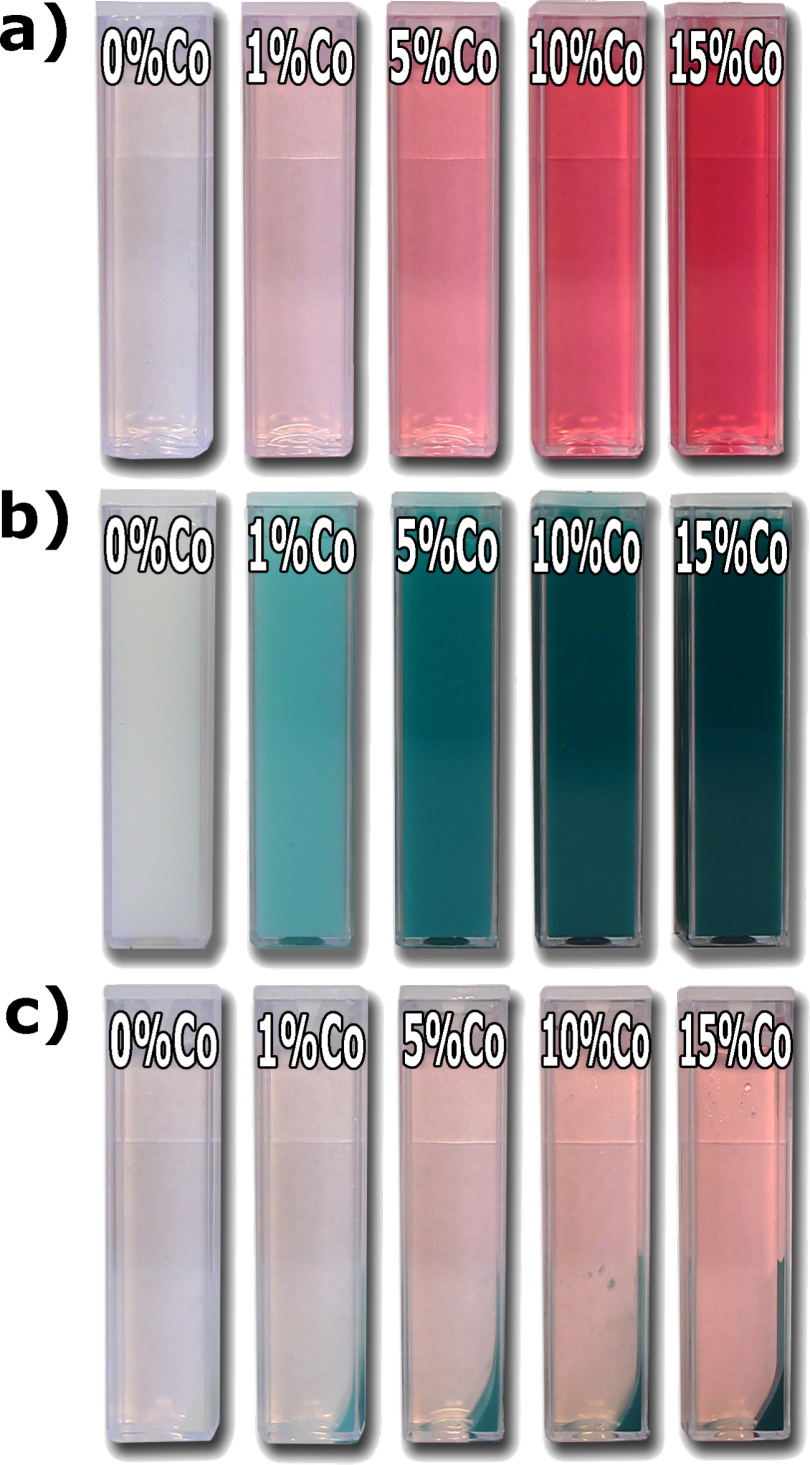
(a) Photographs of the Zn_1−_*_x_*Co*_x_*O precursor in ethylene glycol where the color varies with the composition. (b) Photographs of nanoparticle dispersions in ethylene glycol after microwave solvothermal synthesis. (c) Photographs of nanoparticle dispersions in ethylene glycol after sedimentation.

The experimentally determined doping level, identified by means of ICP-OES, as a function of the concentration of Co^2+^ ions in solution (nominal concentration) is shown in Figure 4 and Table 1. The actual doping level is approximately 10% lower than the Co concentration in the precursor solution. The difference between the theoretical and the measured concentration of cobalt in the Zn_1−_*_x_*Co*_x_*O samples is due to the incomplete degree of reaction of substrates during the synthesis of Zn_1−_*_x_*Co*_x_*O. The pink color of the solution (Figure 5c) after sedimentation confirms that not all the cobalt in solution was consumed during the synthesis reaction. Nevertheless, in this paper, the nominal concentration was used to identify the samples. The color of synthesized Zn_1−_*_x_*Co*_x_*O varied from pure white (undoped), to light green, to dark green, as the Co composition increased from 0 to 15 mol % (Figure 5).

**Table 1:** Results of the chemical analysis of Co-doped ZnO nanopowders for the samples before annealing.

Sample	Measured dopant content	
	ICP-OES	
	Zn (mol %)	Co (mol %)

Zn_0.99_Co_0.01_O	99.13	0.87
Zn_0.95_Co_0.05_O	95.50	4.50
Zn_0.90_Co_0.10_O	90.86	9.14
Zn_0.85_Co_0.15_O	86.40	13.60

**Figure 6:**
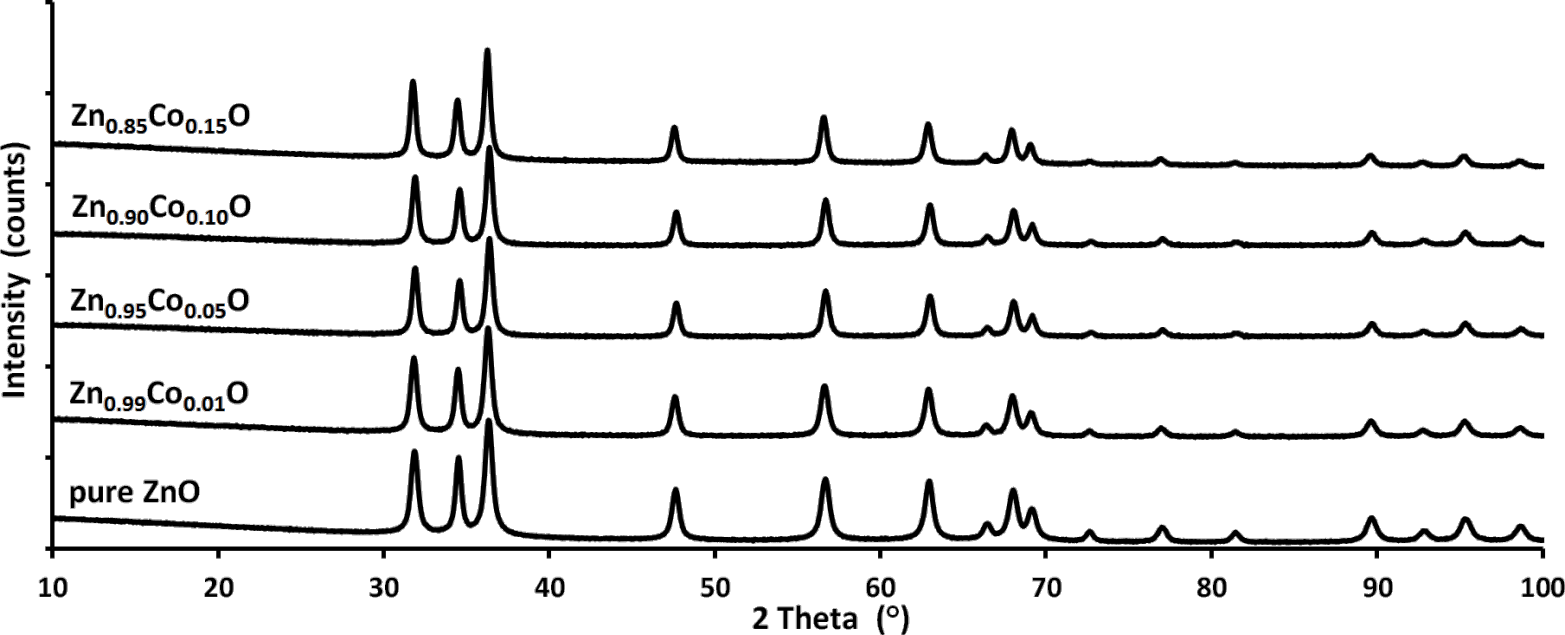
XRD patterns for Zn_1−_*_x_*Co*_x_*O nanopowders before annealing, with a nominal Co content in solution of 0, 1, 5, 10, and 15 mol %.

**Figure 7:**
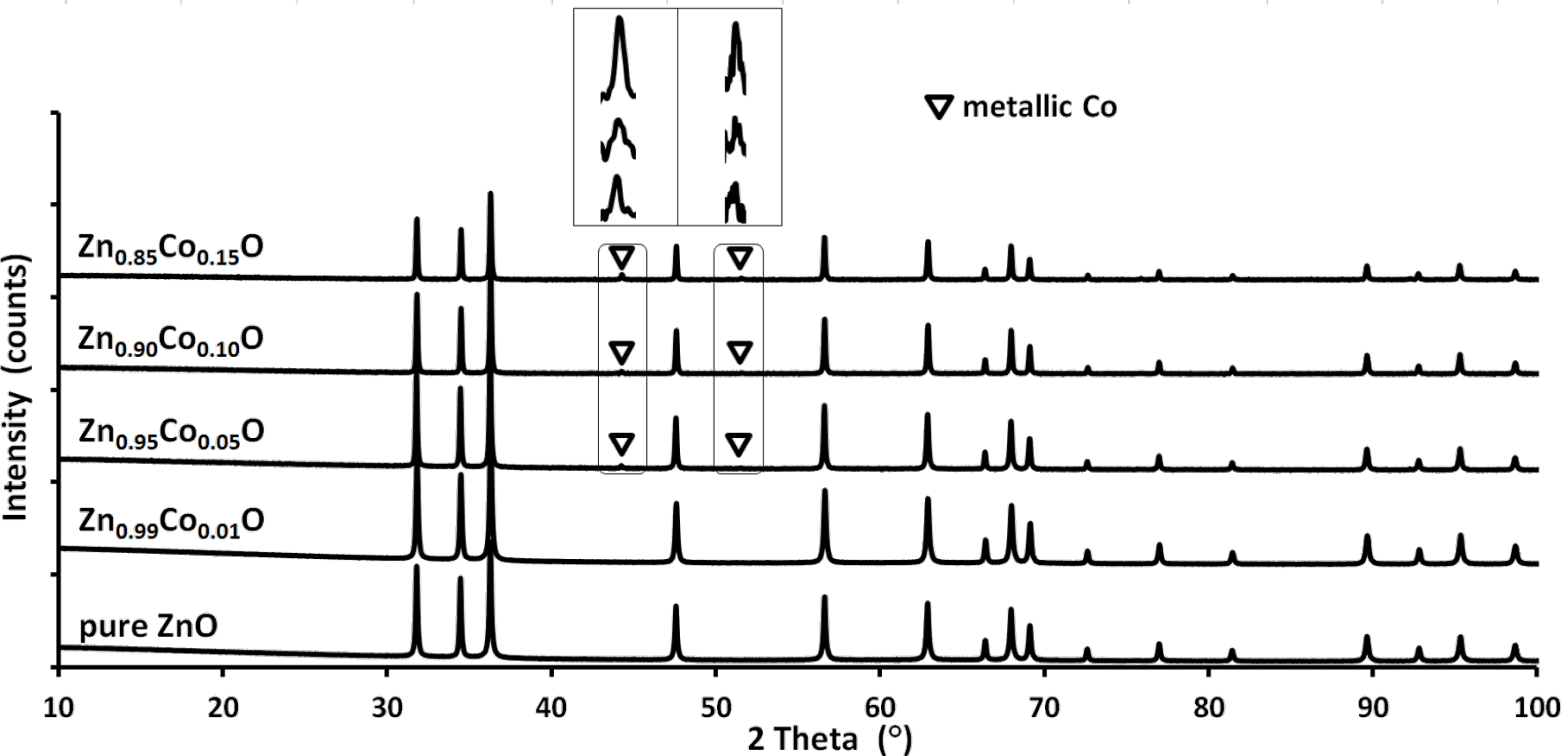
XRD patterns for Zn_1−_*_x_*Co*_x_*O nanopowders after annealing at 800 °C in nitrogen. The metallic α-Co phase can be seen for 5, 10 and 15 mol % samples. The triangle indicates peaks from the metallic α-Co phase.

**Figure 8:**
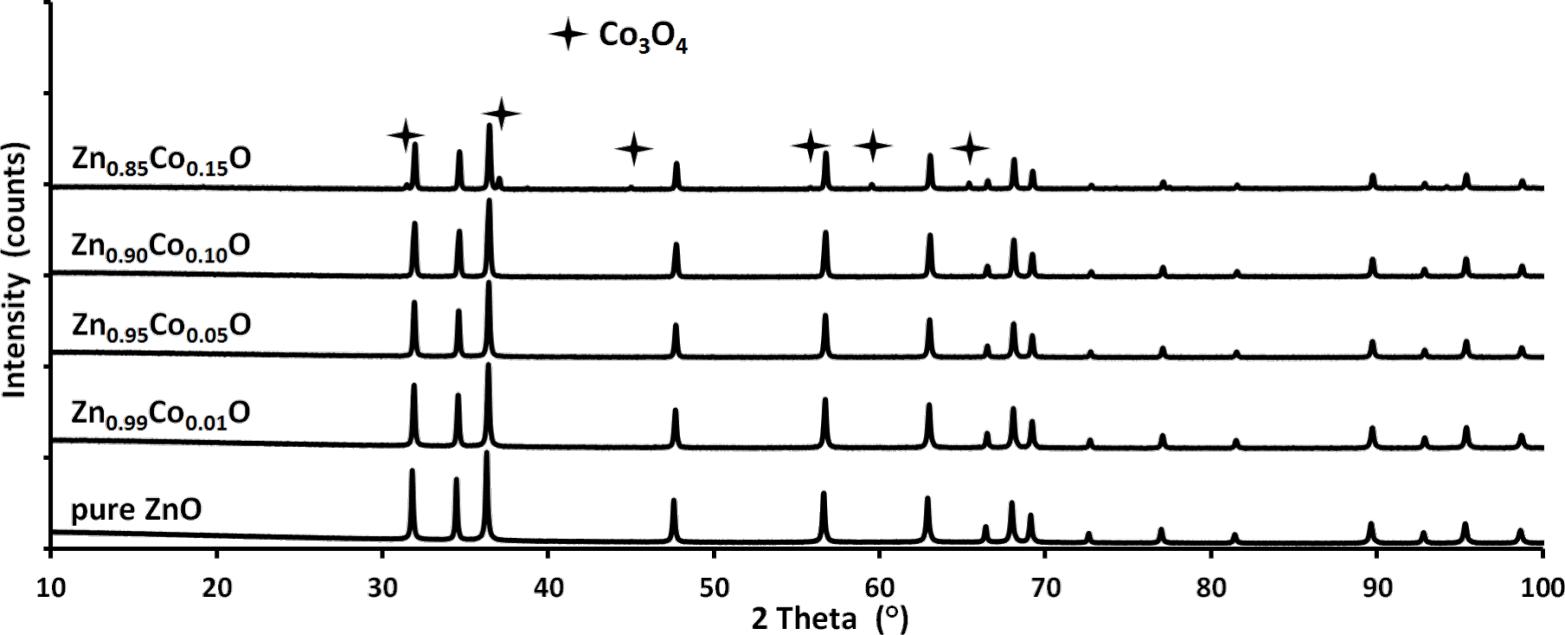
XRD patterns for Zn_1−_*_x_*Co*_x_*O nanopowders after annealing at 800 °C in synthetic air. The Co_3_O_4_ phase can be seen for the 15 mol % Co ion sample. The star indicates peaks assigned to the Co_3_O_4_ phase.

The XRD results for the nanopowders directly after synthesis and after annealing in nitrogen and synthetic air are presented in Figure 6, Figure 7 and Figure 8, respectively. For the materials which were not annealed, the XRD patterns show a single phase tendency of the samples with a hexagonal wurtzite structure. Peaks associated with cobalt oxide or cobalt hydroxide phases such as CoO, Co_2_O_3_ or Co(OH)_2_ were not detected. As confirmed in Figure 7, annealing at 800 °C in nitrogen atmosphere results in the visible precipitation of metallic α-cobalt for the samples with more than 1 mol % Co ion content [72]. When annealed in synthetic air, some precipitation of Co_3_O_4_ was observed in the XRD investigations for the 15 mol % sample only (Figure 8) [72].

**Figure 9:**
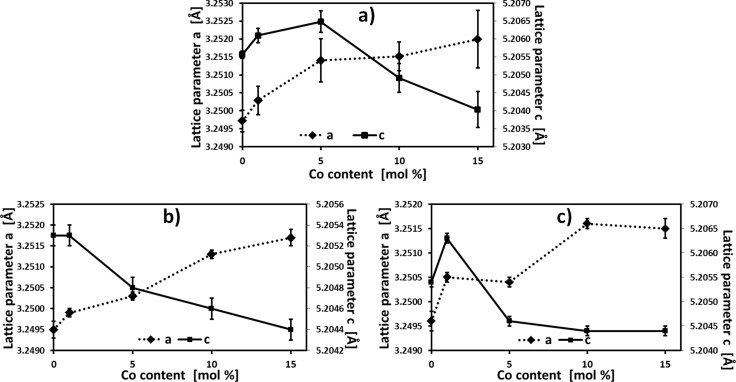
Dependence of the *a* and *c* lattice parameter on nominal Co content, in mol % for: (a) preannealed samples, (b) after annealing in nitrogen, and (c) after annealing in synthetic air.

The wurtzite structure of ZnO has a hexagonal unit cell with two lattice parameters, *a* and *c*. The lattice parameters of ZnO are *a* = 3.2495 Å and *c* = 5.2069 Å; their ratio *c*/*a* = 1.6024 is close to the value for the hexagonal close-packed (hcp) crystal structure in the ratio of *c*/*a* = 1.6330. CoO has a face-centered cubic structure characterized by a lattice parameter of 4.260 Å. To calculate the *c* and *a* lattice parameters, the Rietveld analysis technique [73–76] was used. Zinc (II+) has an ionic radius of 0.74 Å whereas cobalt (II+) has 0.745 Å; therefore, no change in lattice parameters is expected when Zn is replaced by Co in the ZnO crystal lattice. We assume, however, that increasing the concentration of the cobalt dopant should change the lattice parameters, which is related to the CoO cubic structure. In fact, the observed change in lattice parameter shown in Table 2 can be explained by lattice distortions. The lattice parameter *a*, for both the pre- and post-annealed sample, increases from 3.249 Å to 3.252 Å with an increase in Co content. The lattice parameter *c* increases from 5.205 Å (in all cases, 0% Co) up to 5.2065 Å for 5 mol % Co for the preannealed sample, and up to 5.2063 Å for 1 mol % Co after annealing in synthetic air. The *c* lattice parameter decreases to ≈5.204 Å with further increase of the Co content. The data shown in Figure 9 and Table 3 present the changes in lattice parameters of the samples, indicative of Co atoms replacing Zn atoms in the ZnO lattice [8]. We also observed a decrease in the *c*/*a* ratio (Table 3), which is caused by the change in size and the proportion of the unit cell.

**Table 2:** Lattice parameters of ZnO and ZnO doped with Co for samples before and after annealing.

Sample	Before annealing		After annealing in nitrogen		After annealing in synthetic air	
	*a* (Å)	*c* (Å)	*a* (Å)	*c* (Å)	*a* (Å)	*c* (Å)

ZnO	3.2497 ± 0.0001	5.2056 ± 0.0003	3.2495 ± 0.0001	5.2053 ± 0.0002	3.2496 ± 0.0001	5.2054 ± 0.0002
Zn_0.99_Co_0.01_O	3.2503 ± 0.0002	5.2061 ± 0.0004	3.2499 ± 0.0001	5.2053 ± 0.0001	3.2505 ± 0.0002	5.2063 ± 0.0004
Zn_0.95_Co_0.05_O	3.2514 ± 0.0003	5.2065 ± 0.0006	3.2503 ± 0.0001	5.2048 ± 0.0001	3.2504 ± 0.0001	5.2046 ± 0.0001
Zn_0.90_Co_0.10_O	3.2515 ± 0.0004	5.2049 ± 0.0004	3.2513 ± 0.0001	5.2046 ± 0.0001	3.2516 ± 0.0001	5.2044 ± 0.0002
Zn_0.85_Co_0.15_O	3.2520 ± 0.0005	5.2040 ± 0.0008	3.2517 ± 0.0001	5.2044 ± 0.0002	3.2515 ± 0.0001	5.2044 ± 0.0002

**Table 3:** Ratio of the lattice parameters for ZnO and ZnO doped with Co for samples before and after annealing.

Sample	Before annealing	After annealing in nitrogen	After annealing in synthetic air	In hcp structure, ZnO
	*c*/*a*	*c*/*a*	*c*/*a*	

ZnO	1.6019	1.6018	1.6019	1.6330
Zn_0.99_Co_0.01_O	1.6017	1.6017	1.6017	
Zn_0.95_Co_0.05_O	1.6013	1.6013	1.6012	
Zn_0.90_Co_0.10_O	1.6008	1.6008	1.6006	
Zn_0.85_Co_0.15_O	1.6003	1.6005	1.6006	

The density of Co-doped ZnO powders after synthesis was ≈5.2 g/cm^3^ and the SSA was ≈38 m^2^/g (Table 3). Based on the assumption of spherical particles and on the SSA measurements, the average particle diameter was calculated (Equation 1) to be 31 nm (Table 4). Based on the Scherrer equation, the size of crystalline particles in powder form was calculated (Equation 2) to be ≈26 nm (Table 4).

[1]
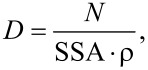


where *D* is the mean particle size (µm), *N* is a factor dependent on the shape of the particles (for spherical particles *N* = 6), SSA is the specific surface area (m^2^/g), and ρ is the density (g/cm^3^). The Scherrer equation,

[2]
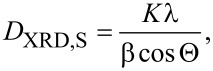


can be used to calculate the mean particle diameter, *D*_XRD,S_, where *K* is a dimensionless shape factor with a value close to unity (the shape factor has a typical value of about 0.9, but varies with the actual shape of the crystallite), λ is the X-ray wavelength, β is the line broadening at half of the maximum intensity (FWHM), and Θ is the Bragg angle.

**Table 4:** Skeleton density and SSA of ZnO and ZnO doped with Co for samples before and after annealing.

Sample	Before annealing		After annealing in nitrogen		After annealing in synthetic air	
	SSA by gas adsorption, *a*_s_ ± σ (m^2^/g)	Skeleton density by gas pycnometry, ρ_s_ ± σ (g/cm^3^)	SSA by gas adsorption, *a*_s_ ± σ (m^2^/g)	Skeleton density by gas pycnometry, ρ_s_ ± σ (g/cm^3^)	SSA by gas adsorption, *a*_s_ ± σ (m^2^/g)	Skeleton density by gas pycnometry, ρ_s_ ± σ (g/cm^3^)

ZnO	39 ± 1	5.20 ± 0.01	3 ± 1	5.64 ± 0.03	3 ± 1	5.64 ± 0.03
Zn_0.99_Co_0.01_O	38 ± 1	5.16 ± 0.02	3 ± 1	5.61 ± 0.04	3 ± 1	5.62 ± 0.03
Zn_0.95_Co_0.05_O	39 ± 1	5.11 ± 0.02	3 ± 1	5.64 ± 0.04	3 ± 1	5.61 ± 0.02
Zn_0.90_Co_0.10_O	37 ± 1	5.16 ± 0.01	3 ± 1	5.64 ± 0.04	3 ± 1	5.60 ± 0.01
Zn_0.85_Co_0.15_O	37 ± 1	5.15 ± 0.02	3 ± 1	5.64 ± 0.02	3 ± 1	5.63 ± 0.02

As shown in Table 5, the results of the grain size calculations obtained with use of BET theory and XRD methods were similar. Such a good agreement of the two values is an indication that the nitrogen used in the SSA experiment easily penetrates between the particles (i.e., they are not sintered to each other). The density of the nanoparticles was lower as compared to a similar conventional material (Table 4). This is because the nanoparticle density is reduced due to a high contribution of the surface layers that were packed less densely than the bulk and include some adsorbed molecules from the ambient atmosphere [77]. The theoretical density for the ZnO micropowder is 5.61 g/cm^3^ [78]. The density of Zn_1−_*_x_*Co*_x_*O NPs decreases following an increase in Co content, which is a result of the lower atomic weight of Co atoms (58.69) as compared to Zn (65.38) as shown in Table 4. The density of Co-doped ZnO powders after annealing was ≈5.6 g/cm^3^, as expected, since the contribution of surface layers to the density becomes negligible for a large grain size. The low SSA of 3 m^2^/g (Table 3) was caused by an increase in crystallite size, which can be observed in the SEM images (Figure 2 and Figure 3). Based on the SSA measurements, after annealing, the average particle diameter was calculated to be 350 nm (Table 4).

**Table 5:** Grain size of undoped nanopowders and those doped with Co for samples before and after annealing.

Sample	Before annealing		After annealing in nitrogen	After annealing in synthetic air
	Average grain size from SSA (BET), *d* ± σ (nm)	Average crystallite size (Scherrer’s formula, based on XRD), *d* (nm)	Average grain size from SSA (BET), *d* ± σ (nm)	Average grain size from SSA (BET), *d* ± σ (nm)

ZnO	30 ± 1	*d*_a_ = 25, *d*_c_ = 28	355 ± 133	355 ± 133
Zn_0.99_Co_0.01_O	31 ± 1	*d*_a_ = 24, *d*_c_ = 26	355 ± 133	350 ± 133
Zn_0.95_Co_0.05_O	30 ± 1	*d*_a_ = 22, *d*_c_ = 23	355 ± 133	351 ± 133
Zn_0.90_Co_0.10_O	32 ± 1	*d*_a_ = 23, *d*_c_ = 24	355 ± 133	353 ± 133
Zn_0.85_Co_0.15_O	32 ± 1	*d*_a_ = 25, *d*_c_ = 23	355 ± 133	355 ± 133

### XAS investigations of Zn_1−_*_x_*Co*_x_*O before annealing

**Figure 10:**
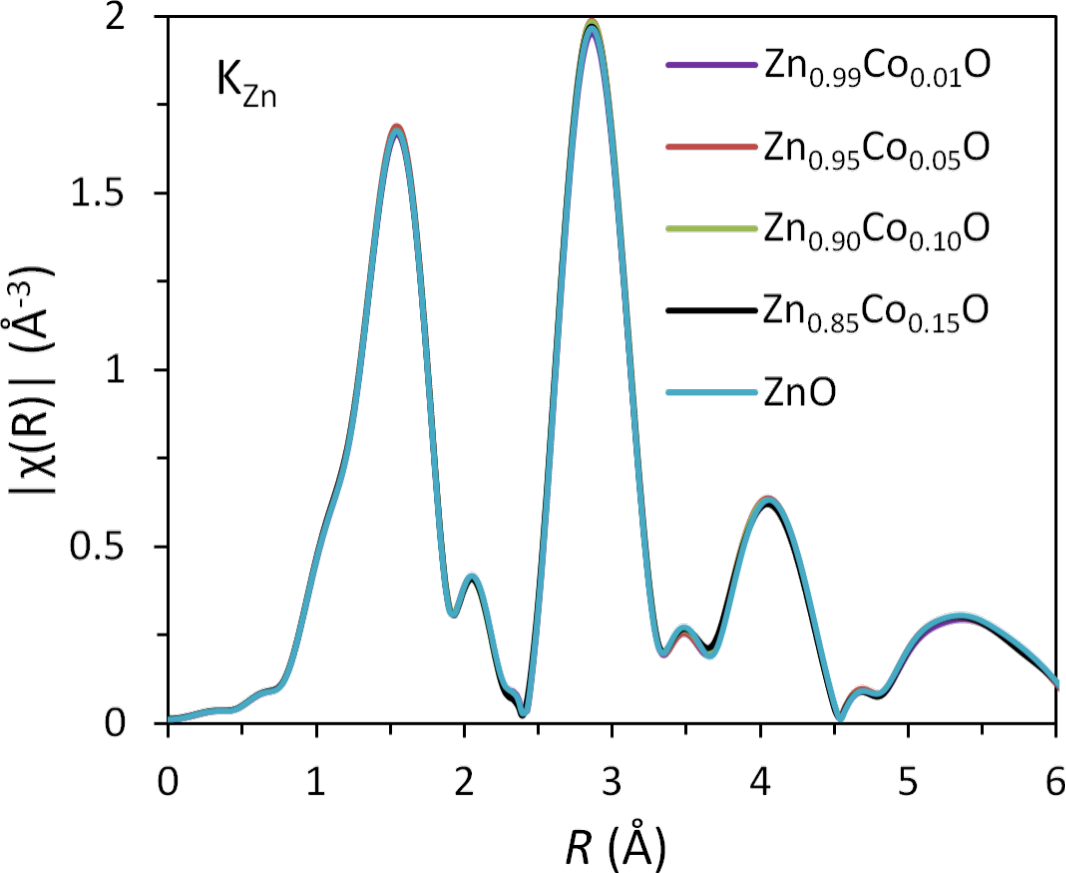
Fourier transformations of EXAFS oscillations at the Zn K-edge for the as-synthesized NP samples.

The data shown in Figure 10 presents the Fourier transformations of EXAFS oscillations taken at the Zn K-edge for the as-synthesized nanopowders. These data indicate that the introduction of Co into ZnO does not cause any structural changes in the investigated samples.

**Figure 11:**
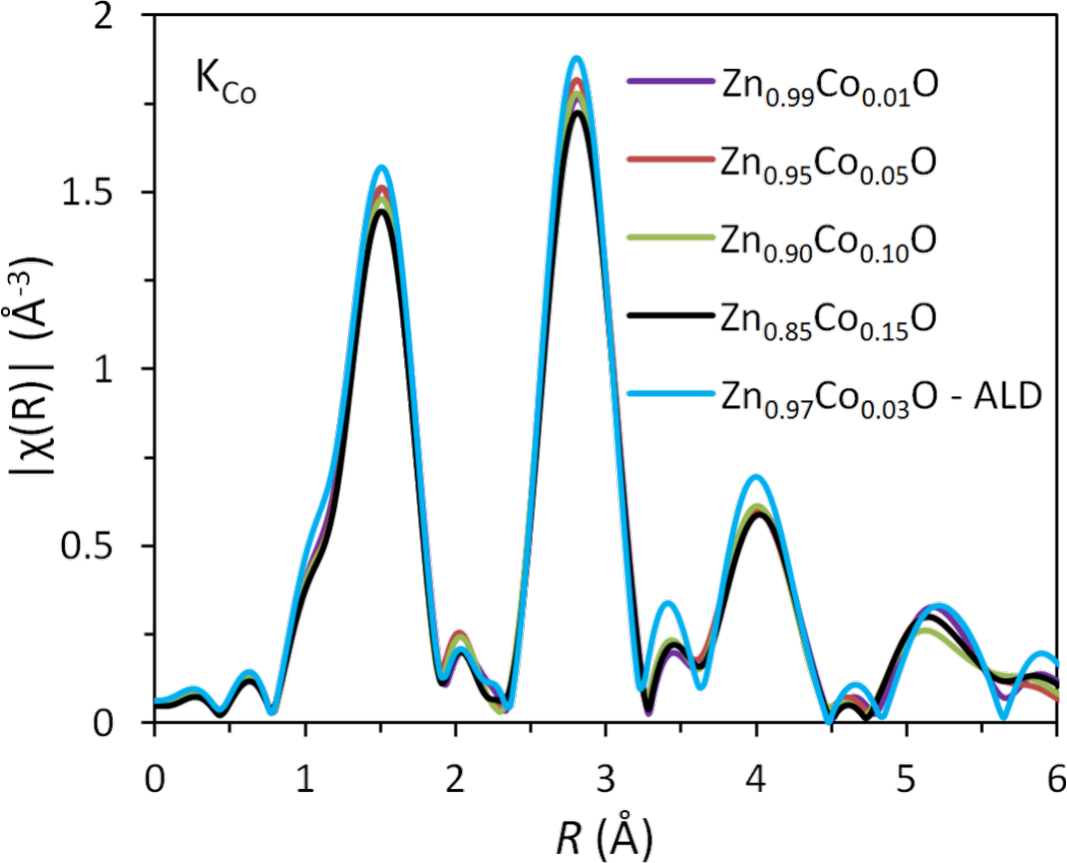
Comparison of the Fourier transformations of EXAFS oscillations measured at the Co K-edge for Zn_1−_*_x_*Co*_x_*O NPs and the reference Zn_1−_*_x_*Co*_x_*O layer grown by ALD for samples before annealing.

A comparison of the Fourier transformations of EXAFS oscillations (measured at the Co K-edge for four types of Zn_1−_*_x_*Co*_x_*O NPs and for the reference Zn_1−_*_x_*Co*_x_*O sample grown by atomic layer deposition (ALD)) is shown in Figure 11. The method of preparation for this sample is described in [42]. For the latter sample, we independently proved that all Co ions introduced into ZnO substitute for Zn, forming uniform Zn_1−_*_x_*Co*_x_*O NPs [41–42].

The data shown in Figure 11 confirmed the Co substitution in all investigated NPs. No metallic Co inclusions were observed. This observation is in line with the magnetic investigations of the produced nanopowders.

### Magnetic investigations of Zn_1−_*_x_*Co*_x_*O

The results of the magnetic properties of the as-synthesized NPs are presented herein. For the as-synthesized samples, a typical paramagnetic response is observed. We did not detect any evidence of a ferromagnetic response. It follows that samples produced using MSS technology are paramagnetic, as was also observed for ZnMnO nanoparticles [34].

A Curie–Weiss temperature, θ, can be estimated by extrapolating the linear part of the χ^−1^(*T*) dependence. The Curie–Weiss temperature is equal to about θ = −78 K. The negative sign of this value suggests a contribution of the antiferromagnetically coupled Co–Co pairs, as often reported for a Zn_1−_*_x_*Co*_x_*O system [41]. The contribution of such pairs to the magnetic response was also observed in electron spin resonance (ESR) investigations of Zn_1−_*_x_*Co*_x_*O bulk samples [79].

**Figure 12:**
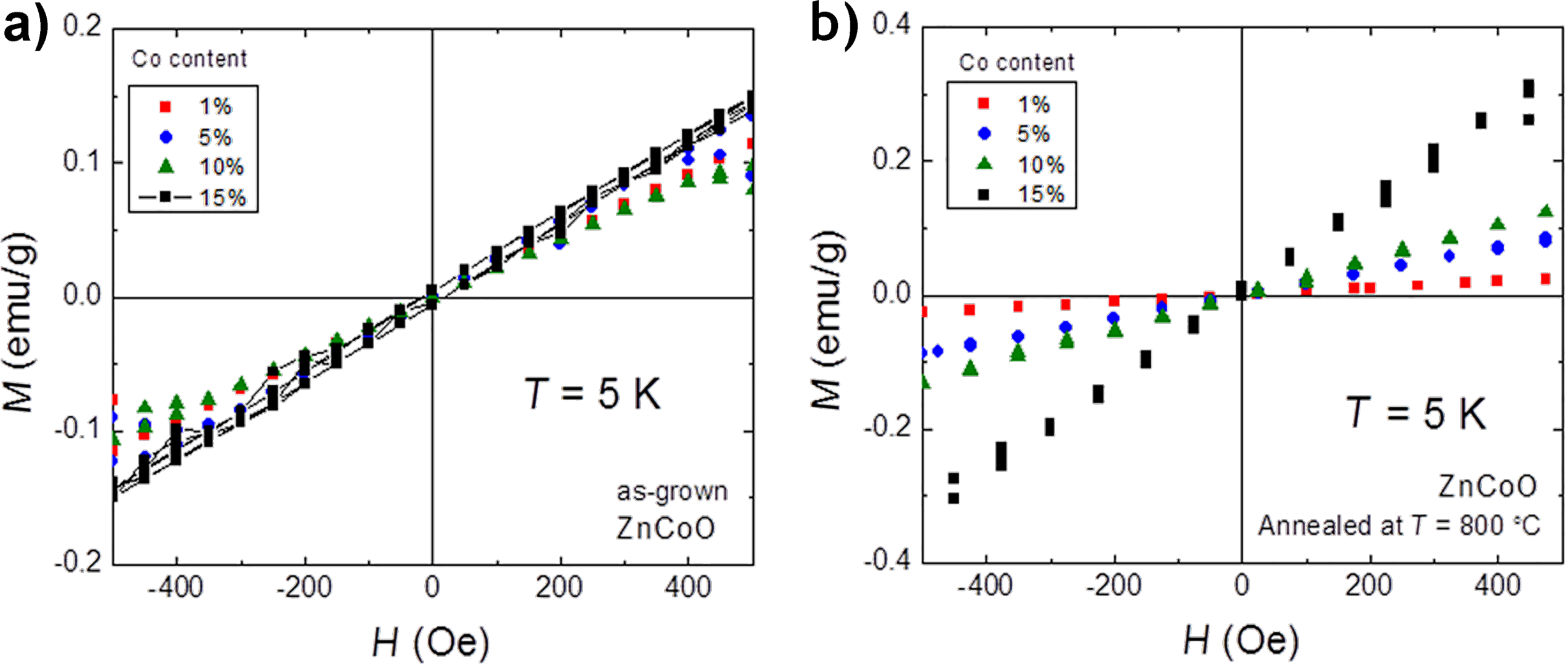
The magnetization dependence on the external magnetic field: (a) for as-synthesized Zn_1−_*_x_*Co*_x_*O NPs with various Co content (in mol %) and (b) for annealed Zn_1−_*_x_*Co*_x_*O NPs in synthetic air with various Co content (in mol %).

Surprisingly, as shown in Figure 12a, the *M*(*H*) measurements reveal nearly the same magnetization up to *H* = 0.5 kOe for samples with different Co fractions, that is, the magnetization does not scale with Co fraction in the samples. Such behavior is in line with the clearly resolved effects of antiferromagnetic coupling of Co–Co pairs. The number of such pairs should increase with an increase in Co concentration. These Co ions do not contribute to the magnetization of the samples since Co–Co coupling is antiferromagnetic.

**Figure 13:**
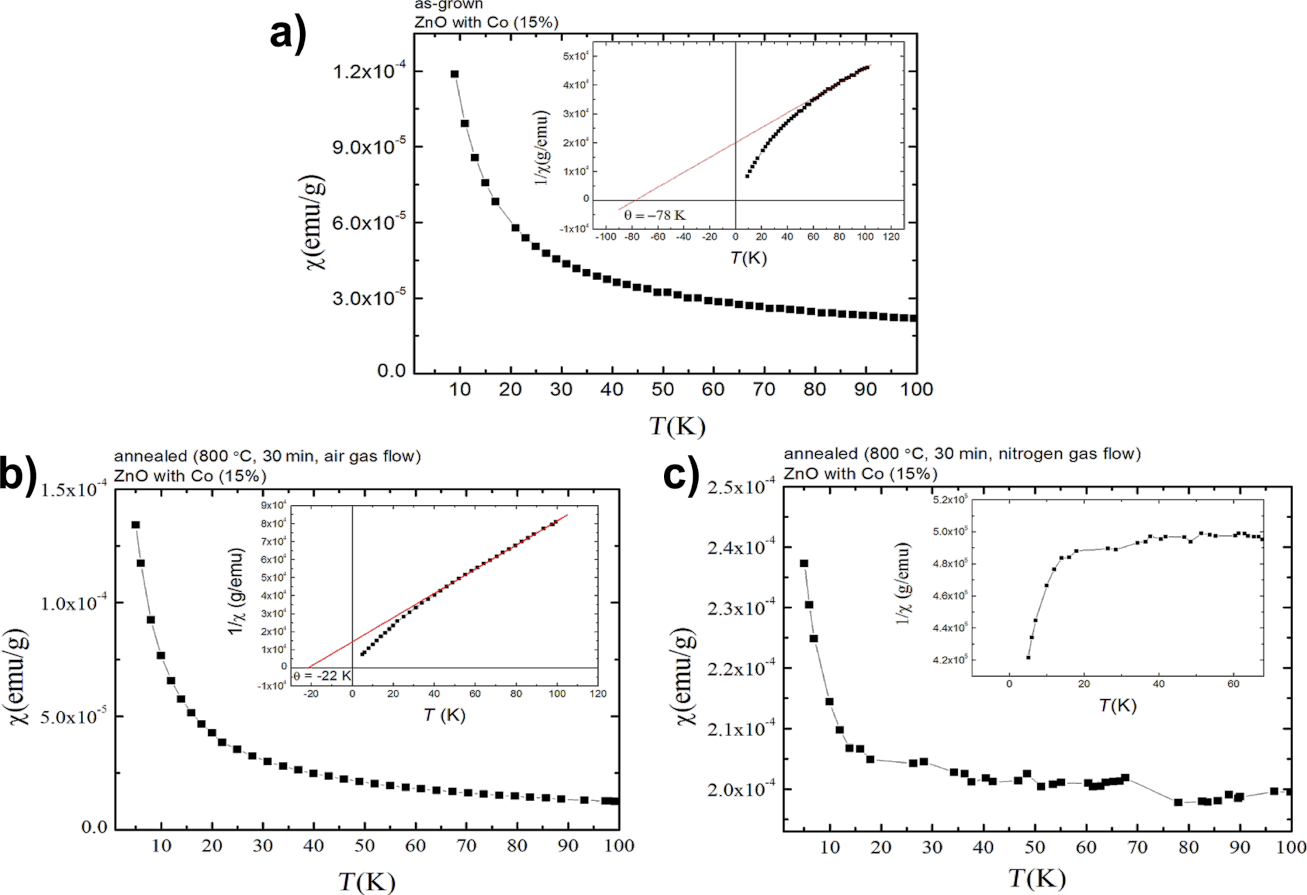
Magnetic susceptibility dependence on temperature for Zn_0.85_Co_0.15_O powder: (a) as-synthesized, b) annealed in synthetic air, and c) annealed in nitrogen. The Curie–Weiss temperature was estimated from 1/χ(*T*) data for an as-synthesized and annealed in synthetic air, Zn_0.85_Co_0.15_O sample. The data were taken for samples with a nominal 15% Co fraction.

Annealing in synthetic air results in similar paramagnetic properties of the samples as obtained for the as-synthesized samples. The paramagnetic response dominates with some contribution of an antiferromagnetic coupling. However, magnetization increases noticeably with an increasing Co content (Figure 12b). Moreover, annealing the antiferromagnetic contribution decreases θ down to about −22 K (Figure 13b).

In Figure 13 we compare the magnetic response for as-synthesized samples (Figure 13a), the samples annealed in synthetic air (Figure 13b) and annealed in high purity nitrogen (Figure 13c). The magnetic response of the as-synthesized and annealed in air samples is qualitatively similar. Two differences are as follows. First, the difference in the Curie temperature can be seen. Secondly, the magnetization increases with the Co fraction increase for the annealed sample.

The situation changes drastically for samples annealed in high purity nitrogen gas (Figure 13c). The reciprocal magnetic susceptibility is only weakly temperature dependent for *T* > 20 K (Figure 13c) and then at low temperature, it drops rapidly to zero at *T* > 0 K. For this material, the XRD investigation shows formation of metallic inclusions. Thus, we assume that the situation closely follows that observed for Zn_1−_*_x_*Co*_x_*O N layers grown by ALD [41]. For the ALD samples grown at a temperature greater than 220 °C, the presence of Co metal inclusions was detected in TEM investigations [41]. For relatively low concentrations, we observed super-paramagnetism only, because even though each inclusion was in a ferromagnetic state, they did not interact with one another. Only when the Co metal inclusions were dense were they coupled and a ferromagnetic response resulted [41]. The present results are in line with that observed for Zn_1−_*_x_*Co*_x_*O films grown by ALD. No ferromagnetic response was detected in a zero magnetic field. Once the field was turned on, the magnetization was nearly saturated and the reciprocal magnetic susceptibility was practically temperature independent.

The results of the magnetic studies for samples annealed at 800 °C may shed light on the origin of often very confusing reports of Zn_1−_*_x_*Co*_x_*O samples. For those prepared using MSS, paramagnetic NPs were obtained. Annealing in the presence of oxygen (even trace amounts) led to formation of various Co oxides, and the paramagnetic behavior was preserved. On the other hand, Co metal inclusions were formed when annealing was performed under a reducing atmosphere, leading to ferromagnetic response.

The sensitivity of the Zn_1−_*_x_*Co*_x_*O NP properties on synthesis conditions may explain the reported difficulties in reproducing the magnetic properties of Zn_1−_*_x_*Co*_x_*O powders and layers.

### Summary of results

For application as magnetic labels or in spintronics, Zn_1−_*_x_*Co*_x_*O NPs should show a ferromagnetic response at room temperature. However, such a response was not present in our prepared samples made using the microwave solvothermal process. In the MSS process, the particles were produced at 220 °C, a relatively low temperature, and the result was NPs with Co substituting for Zn. Such samples are paramagnetic. Annealing in a nitrogen (reducing) atmosphere leads to precipitation of metallic Co inclusions. The presence of such inclusions leads to a ferromagnetic response [80–81]. Samples annealed up to 800 °C in an oxygen containing atmosphere remain paramagnetic. Thus, annealing in different gasses shows quite different magnetic responses. These different responses can be related to the Co metal inclusions and the formation of various foreign phases.

## Conclusion

The microwave solvothermal synthesis allows for the preparation of a uniform, nanocrystalline Zn_1−_*_x_*Co*_x_*O sample with a high Co concentration of up to 15 mol %. The Zn_1−_*_x_*Co*_x_*O NPs have a fully pure, single phase, wurtzite, crystalline structure corresponding to zinc oxide. No other secondary phase such as Co(OH)_2_, CoO, Co_3_O_4_ or Co metal was found for *x* ≤ 0.15, which shows that the doped Co ions are substituted at the Zn ion sites. As shown, the MSS method produced Zn_1−_*_x_*Co*_x_*O NPs samples of very high purity, as demonstrated by XRD and EXAFS analysis. The average grain size of the Zn_1−_*_x_*Co*_x_*O NPs was in the 30 nm range and the shape of the Zn_1−_*_x_*Co*_x_*O NPs was spherical. There was no effect due to the change in the morphology caused by increasing the cobalt dopant.

Zn_1−_*_x_*Co*_x_*O NPs produced at low temperature (220 °C) by the MSS method are paramagnetic for Zn_1−_*_x_*Co*_x_*O in the range of 0–15 mol %. Annealing at 800 °C in nitrogen causes the formation of metallic inclusions, while their annealing in artificial air preserves the paramagnetic properties. The obtained results clearly show that quite different magnetic responses of Zn_1−_*_x_*Co*_x_*O NP samples may result depending on the synthesis conditions and post-growth processing. However, the Zn_1−_*_x_*Co*_x_*O NPs with Co substituted for Zn is paramagnetic with some antiferromagnetic coupling.

## Experimental

### Preparation of Zn_1−_*_x_*Co*_x_*O nanopowders

A solid mixture with concentration of 1, 5, 10 and 15 mol % of Co(CH_3_COO)_2_·4H_2_O (pure for analysis) in Zn(CH_3_COO)_2_·2H_2_O (pure for analysis) was dissolved in ethylene glycol (EG, pure, Chempur). The metal acetates were purchased from Sigma-Aldrich and Chempur and used without further purification. Zinc oxide was obtained by microwave solvothermal synthesis (MSS) [68]. MSS permits rapid and uniform heating, and synthesis under high purity conditions in a closed vessel with precise control of the reaction time. The microwave-driven reaction was conducted in a teflon vessel in a Magnum II reactor (Ertec, Poland) at 220 °C. EG is an excellent absorber of microwave radiation. The reaction duration for all experiments was 25 min under a constant pressure of 0.1 MPa at a microwave power of 600 W. At the end of the reaction, the precipitate was sedimented, washed two times with deionized water and ethanol, centrifuged and dried in a laminar chamber for 24 h. Magnetic investigations were performed both for as-produced dried powders and after annealing at 800 °C for 0.5 h in high purity (99.999%) nitrogen (Messer, Poland) and synthetic air (Multax, Poland) in a tube furnace (PR-60/1200, PIE, Poland).

### X-ray powder diffraction analysis

The X-ray diffraction (XRD) patterns were collected in the 2Θ range of 20–100° at room temperature, with a step increment of 0.02° using an XRD diffractometer (X’Pert PRO diffractometer, Cu Kα radiation; PANalytical BV, Almelo, The Netherlands). Based on the XRD patterns, the average crystallite size was determined using the Scherrer’s formula [82], and lattice parameters were calculated using the Rietveld method.

### Skeletal density and SSA measurements

Skeletal density measurements were carried out using a helium pycnometer (AccuPyc II 1340, Micromeritics, USA) using an in-house procedure [83]. This method enabled the skeletal density of ZnO nanopowders to be measured with an accuracy of 0.01 g/cm^3^. The SSA of the powders was measured by gas adsorption and analyzed with BET theory (Gemini 2360, Micromeritics, USA). The powders were subjected to desorption at 150 °C for 2 h prior to the measurement [69]. The measurement of the average diameter of the particles was taken based on the SSA, density, and the assumption that all particles were spherical and identical [68,83].

### Morphology analysis and elemental composition of samples

The nanopowders were sprayed with a thin layer of carbon using a sputter coater (SCD 005/CEA 035, BAL-TEC, Switzerland). The morphology of the nanopowders was investigated with SEM (Ultra Plus; Carl Zeiss Meditec AG, Jena, Germany).

### Chemical composition of the powders

The experimentally measured ion content in the powders may not be identical to that in the solutions. The chemical composition analysis of powders was examined by inductively coupled plasma optical emission spectrometry (ICP-OES) with induction in argon plasma (Thermo Scientific, iCAP 6000 series, United Kingdom).

The samples for analysis with ICP-OES were prepared as follows: 5 mg of powder was weighed in a 110 mL teflon vessel and 15 mL of deionized water (HLP 20UV, Hydrolab, Poland) was added. Then, 6 mL of HNO_3_ was added and the solution was subjected to one microwave heating cycle in the Magnum II reactor. After cooling, the sample volume was filled to 50 mL with deionized water.

### EXAFS investigations of Zn_1−_*_x_*Co*_x_*O

The local atomic structure of Co in Zn_1−_*_x_*Co*_x_*O NPs with concentrations of 1%, 5%, 10% and 15% Co were determined from the extended X-ray absorption fine structure (EXAFS) measurements. EXAFS measurements at the K-edge of Zn and Co were performed at Deutsches Elektronen-Synchrotron (DESY), Hasylab (C1 and A1 stations) at 140 °C using a 7-element silicon fluorescence drift detector for the Co K-edge and in transmission mode for the Zn K-edge. The data were compared with two types of reference samples: ZnO NPs and Zn_1−_*_x_*Co*_x_*O layers grown by ALD, studied separately [84].

### Magnetic investigations

The measurement of the magnetic properties of both as-grown and annealed Zn_1−_*_x_*Co*_x_*O NPs were performed by a custom-built SQUID magnetometry experiment for the temperature range *T* = 5–200 K at an external magnetic field of up to *H* = 0.5 kOe.
